# Surgical Consideration in Total Knee Arthroplasty for a Patient With Incidental Femoral Intramedullary Fibrous Dysplasia

**DOI:** 10.7759/cureus.79528

**Published:** 2025-02-23

**Authors:** Ren Yi Kow, Suraya Zainul-Abidin, Woon Theng Lo, Xiu Fen Chen, Ming Han Lincoln Liow

**Affiliations:** 1 Department of Orthopaedics, Traumatology and Rehabilitation, International Islamic University Malaysia, Kuantan, MYS; 2 Department of Orthopaedic Surgery, Singapore General Hospital, Singapore, SGP; 3 Department of Anatomical Pathology, Singapore General Hospital, Singapore, SGP

**Keywords:** bone biopsy, bone tumour, fibrous dysplasia (fd), knee osteoarthritis (koa), total knee arthroplasty (tka)

## Abstract

Fibrous dysplasia (FD) is an uncommon, benign bone disorder caused by a somatic mutation in the GNAS gene on chromosome 20, leading to impaired osteoblastic differentiation and the replacement of normal bone with structurally weak, fibro-osseous tissue. It is typically diagnosed during childhood, though some cases remain undetected until incidentally discovered in adulthood.

We present a patient with symptomatic knee osteoarthritis and an incidental finding of monostotic femur FD. The presence of FD in this anatomical location complicates an otherwise straightforward total knee arthroplasty (TKA). A bone biopsy was performed to rule out malignant transformation of the FD. The resulting bone defect posed a risk of periprosthetic fracture due to the stress riser created by the biopsy. A long femoral stem was used to mitigate this risk, and the patient recovered fully.

This case underscores the critical importance of meticulous planning in managing patients with incidental findings of potential bone tumors. Biopsy site selection must consider definitive surgical management while avoiding pitfalls, such as periprosthetic fractures that may arise from stress risers created by biopsy site fenestration.

## Introduction

Fibrous dysplasia (FD) is a rare, benign intramedullary bony lesion characterized by the replacement of normal lamellar bone with fibrous connective tissue and irregular trabecular bone [1-5]. It results from a mutation in the GNAS gene on chromosome 20, leading to failure to form normal lamellar bone, which remains as woven bone. It can be classified as either monostotic or polyostotic, depending on the number of affected sites. Approximately 70% of FD cases are monostotic, involving a single bone [6-8]. Polyostotic FD, on the other hand, can sometimes be associated with McCune-Albright syndrome, which occurs in an estimated one out of 100,000 individuals. Patients with this syndrome commonly present with polyostotic FD, café-au-lait spots, and endocrine abnormalities such as hyperthyroidism, precocious puberty, Cushing syndrome, excess growth hormone, and hypophosphatemia [1,2].

Most patients are diagnosed during childhood following trauma; however, some asymptomatic cases may remain undetected until adulthood. While many patients are asymptomatic and diagnosed incidentally, others may present with localized pain in the affected area. In some cases, complications such as pathological fractures may serve as the initial presentation [8]. In this case report, we present an incidental finding of monostotic FD in a patient undergoing total knee arthroplasty (TKA) for knee osteoarthritis.

## Case presentation

**Figure 1:**
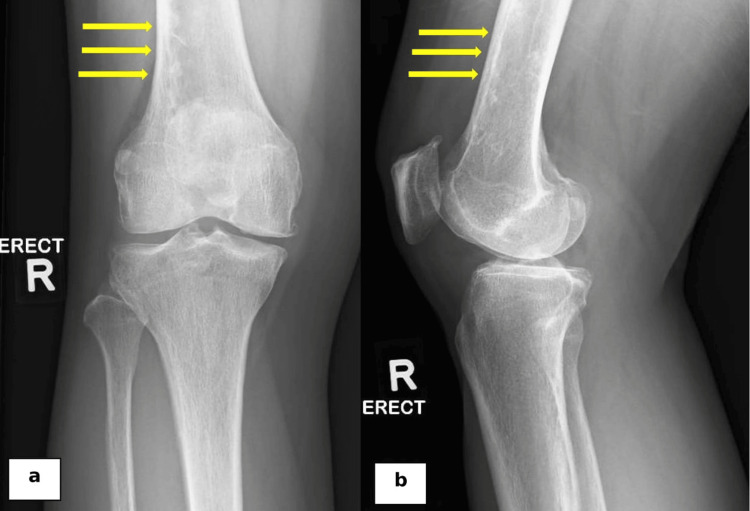
Plain radiographs of the knee in anteroposterior (a) and lateral (b) views revealed tri-compartmental osteoarthritic changes along with a suspicious elongated lucent medullary lesion in the femoral shaft (yellow arrows).

**Figure 2:**
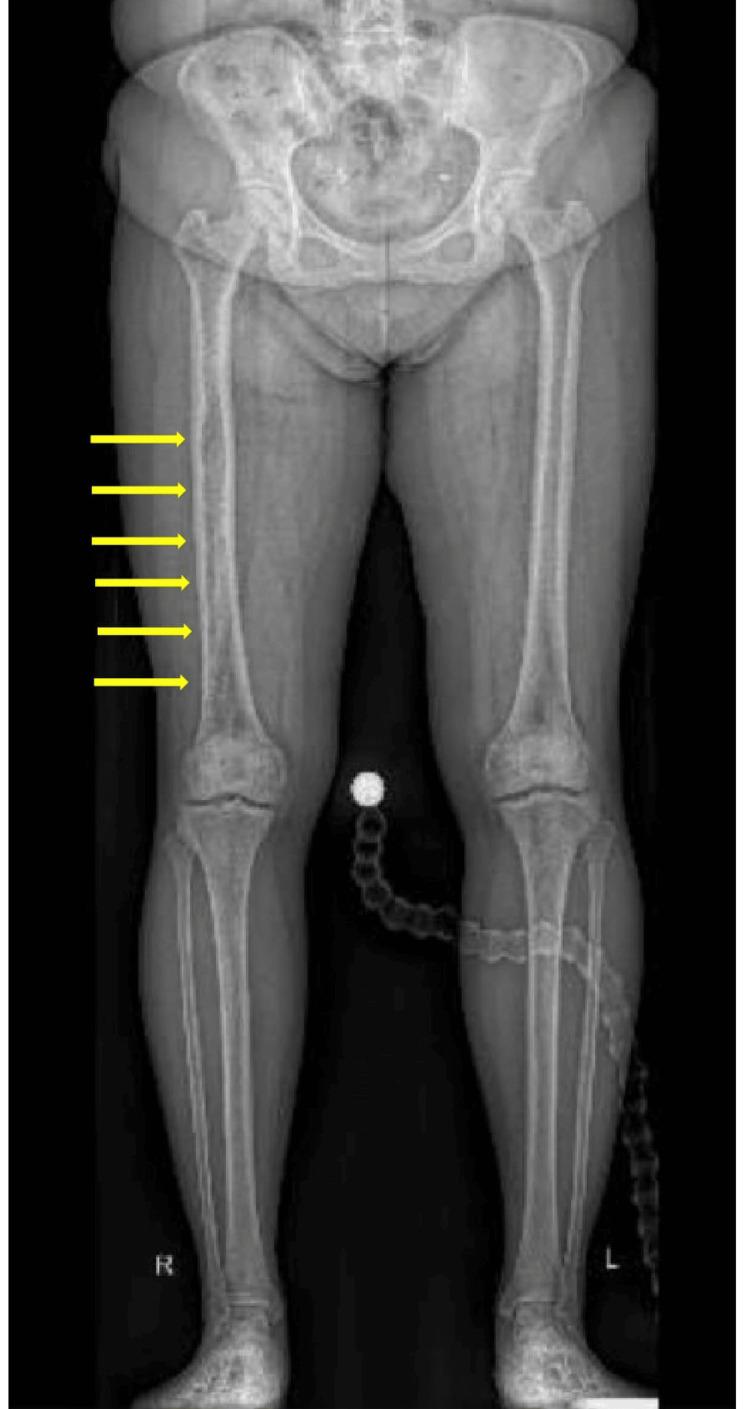
The lower limb plain radiograph delineated an ill-defined lucent intramedullary lesion in the right distal femur, extending to the proximal one-third of the femur (yellow arrows).

A 56-year-old woman presented with worsening right knee pain for one year. She denied experiencing thigh pain and did not report any constitutional symptoms or red flags. There were no indications of endocrine abnormalities. Her knee symptoms did not improve with conservative management. A plain radiograph of the knee revealed tricompartmental osteoarthritic changes along with a suspicious elongated lucent medullary lesion in the femoral shaft (Figure 1). A lower limb radiograph further delineated an ill-defined lucent intramedullary lesion in the right distal femur, extending to the proximal one-third of the femur (Figure 2). No periosteal reaction or pathological fracture was observed.

**Figure 3:**
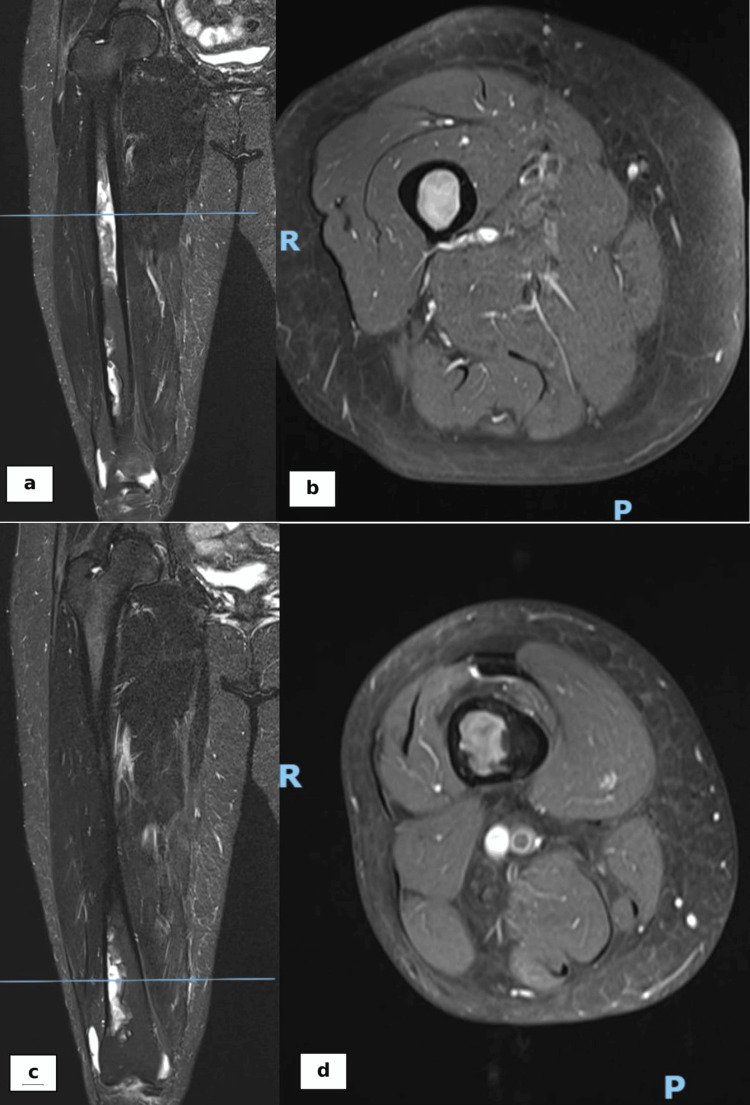
Proximal one-third of the femur in coronal (a) and axial (b) views of the MRI revealed a prominent fatty signal in the femoral shaft without endosteal scalloping, raising suspicion of an intramedullary lipomatous tumor. Similar features were seen on the distal femur in coronal (c) and axial (d) views.

The MRI revealed a prominent fatty signal in the femoral shaft without endosteal scalloping, raising suspicion of an intramedullary lipomatous tumor (Figure 3). A bone scan showed mild increased tracer activity localized to the right femur, with no abnormal tracer uptake at other skeletal sites.

**Figure 4:**
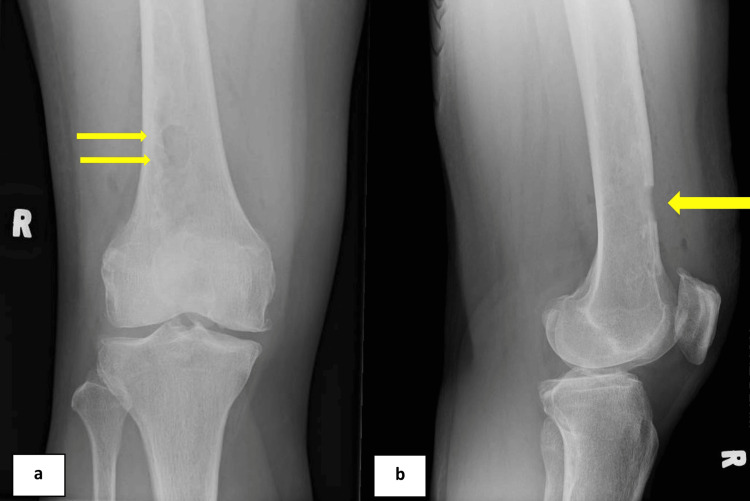
Plain radiographs in anteroposterior (a) and lateral (b) views show the anterior femoral cortical window (yellow arrows) created 9 cm from the joint line to obtain a tissue sample for histopathological examination.

**Figure 5:**
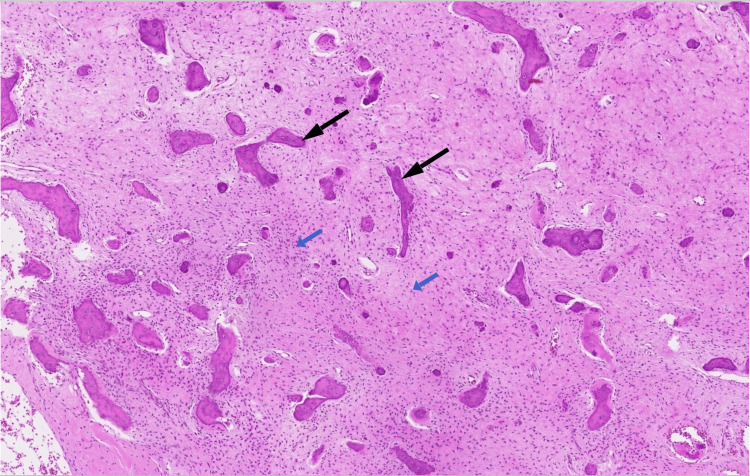
The haematoxylin and eosin sections with 20x magnification (a) and 50x magnification (b) show a fibro-osseous lesion characterised by branching and trabeculae of woven bone without osteoblastic rimming (black arrows), within a fibrous stroma containing bland spindle cells (blue arrows).

Although intraosseous liposarcoma is very rare, its possibility warranted a biopsy to establish a definitive diagnosis. Using an anterolateral approach, an anterior femoral cortical window was created 9 cm from the joint line to obtain a tissue sample for histopathological examination (Figure 4). The biopsy sample was reported as a fibro-osseous lesion consistent with FD (Figure 5).

**Figure 6:**
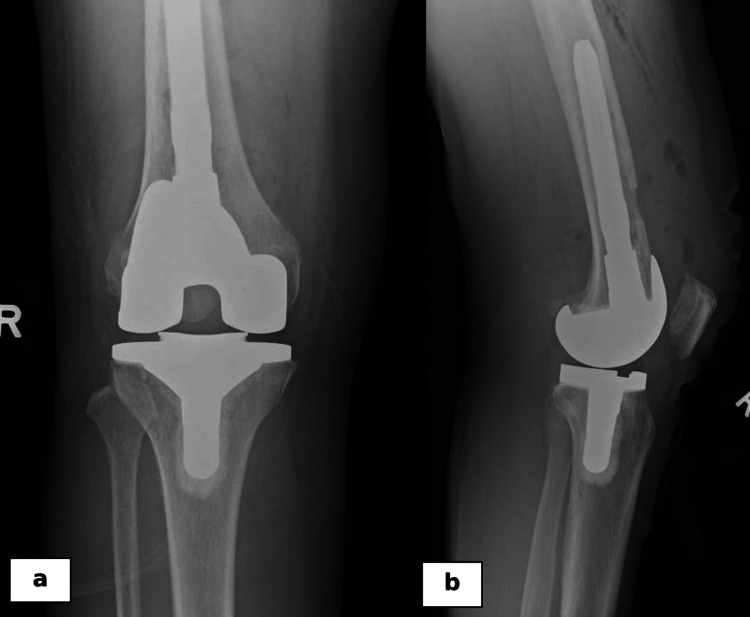
Postoperative plain radiographs of the knee in anteroposterior (a) and lateral (b) views show the long-stem femoral component that was used to bypass the cortical window in the femoral anterior cortex.

Since the lesion was confirmed to be benign, we proceeded with the planned TKA. To address the stress riser created by the bone biopsy, a long-stem femoral component was used to bypass the cortical window in the femoral anterior cortex (Figure 6). The patient recovered well and was able to return to work one month after the procedure.

## Discussion

Total knee arthroplasty is a highly effective surgical procedure for severe knee osteoarthritis, providing pain relief, improved joint function, and enhanced quality of life. Over 90% of patients report significant benefits, and TKA also positively impacts psychosocial well-being by restoring mobility and independence. Most implants have a lifespan of 15 to 20 years, contributing to consistently high patient satisfaction [9-11].

In this patient, the chief complaint was refractory knee pain secondary to advanced knee osteoarthritis. Total knee arthroplasty was deemed appropriate to alleviate her pain and improve her quality of life [11]. However, the incidental finding of a suspicious, potentially malignant, intramedullary bony lesion complicated what would have otherwise been a straightforward surgery. Monostotic FD was diagnosed through a combination of bone scan, MRI, and bone biopsy. A bone biopsy was performed to rule out the possibility of an osseous liposarcoma tumor and to exclude the malignant transformation of a previously benign FD lesion [9]. Notably, Okuda et al. previously reported a case of undifferentiated pleomorphic sarcoma arising in FD, confirmed by GNAS mutation analysis [12].

The selection of the biopsy site to obtain a tissue sample is crucial for subsequent surgical treatment. The biopsy site should account for the location of the definitive incision scar while optimizing the biopsy yield. An anterolateral site was chosen, considering both the definitive TKA scar and the distal femur replacement scar. An anterior cortical window was created 9 cm from the joint line to obtain samples from both the midshaft and distal femur marrow. The window was positioned to avoid being too close to the joint line, which would risk missing the midshaft sample, or too proximal, which would compromise the length needed for a long stem.

The fenestration in the anterior femoral cortex created by the bone biopsy posed a significant risk of stress-riser-induced fracture, particularly due to the biomechanical load placed on the distal femur after TKA. This fenestration at the anterior femoral cortex is analogous to a severe notching. A systematic review by Stamiris et al. demonstrated that anterior femoral notching of 3 mm or more is associated with an increased risk of supracondylar periprosthetic femoral fractures following TKA [13]. To mitigate this risk, a long-stem femoral component was utilized. By bypassing the defect created by the bone biopsy, the long stem facilitated enhanced load sharing and improved implant stability, significantly reducing the likelihood of periprosthetic fracture.

## Conclusions

This case underscores the critical importance of thoroughly evaluating each patient, especially when incidental findings may impact surgical planning. For patients with potential bone tumors, performing a biopsy is essential to rule out malignancy. However, such diagnostic interventions must be carefully planned to minimize their impact on subsequent surgical procedures. In this case, the tumour biopsy created a stress riser, necessitating the use of a long femoral stem to reduce the risk of periprosthetic fracture. This case demonstrates that with meticulous evaluation and a well-thought-out surgical plan, excellent outcomes can be achieved, even in the presence of an incidental bone tumour.
